# Build-up functionalization of anti-EGFR × anti-CD3 bispecific diabodies by integrating high-affinity mutants and functional molecular formats

**DOI:** 10.1038/s41598-020-61840-3

**Published:** 2020-03-18

**Authors:** Ryutaro Asano, Katsuhiro Hosokawa, Shintaro Taki, Shota Konno, Ippei Shimomura, Hiromi Ogata, Mai Okada, Kyoko Arai, Masayoshi Onitsuka, Takeshi Omasa, Takeshi Nakanishi, Mitsuo Umetsu, Izumi Kumagai

**Affiliations:** 1 Department of Biomolecular Engineering, Graduate School of Engineering, Tohoku University, Sendai, 980-8579 Japan; 2 Present Address: Department of Biotechnology and Life Science, Graduate School of Engineering, Tokyo University of Agriculture and Technology, Tokyo, 184-8588 Japan; 3 Institute of Technology and Science, Tokushima University, Tokushima, 770-8506 Japan

**Keywords:** Antibody generation, Protein design, Molecular medicine

## Abstract

Designing non-natural antibody formats is a practical method for developing highly functional next-generation antibody drugs, particularly for improving the therapeutic efficacy of cancer treatments. One approach is constructing bispecific antibodies (bsAbs). We previously reported a functional humanized bispecific diabody (bsDb) that targeted epidermal growth factor receptor and CD3 (hEx3-Db). We enhanced its cytotoxicity by constructing an Fc fusion protein and rearranging order of the V domain. In this study, we created an additional functional bsAb, by integrating the molecular formats of bsAb and high-affinity mutants previously isolated by phage display in the form of Fv. Introducing the high-affinity mutations into bsDbs successfully increased their affinities and enhanced their cytotoxicity *in vitro* and *in vivo*. However, there were some limitations to affinity maturation of bsDb by integrating high-affinity Fv mutants, particularly in Fc-fused bsDb with intrinsic high affinity, because of their bivalency. The tetramers fractionated from the bsDb mutant exhibited the highest *in vitro* growth inhibition among the small bsAbs and was comparable to the *in vivo* anti-tumor effects of Fc-fused bsDbs. This molecule shows cost-efficient bacterial production and high therapeutic potential.

## Introduction

Conventional monoclonal antibodies have been widely used to treat diseases with difficulties to cure such as rheumatism^1,2^. However, particularly in the field of cancer treatments, animal and clinical studies have highlighted the limitations of therapeutic efficacy of monoclonal antibodies^3^. Therefore, many strategies for improving the function of antibodies have been explored; one such strategy is the design of non-natural antibody formats, particularly bispecific antibodies (bsAbs) and antibody fusion proteins, such as immunotoxins. The United States Food and Drug Administration (FDA) has only approved two non-natural antibody designs to treat cancer: blinatumomab in 2009^3^ and moxetumomab pasudotox in 2018^4^. Thus, additional studies are needed to produce next-generation antibody drugs with high therapeutic potential.

BsAbs have ability to simultaneously bind two different targets. For example, bsAbs can redirect various immune cells, mainly cytotoxic T cells and natural killer cells, toward cancer cells. The difficulty in mass production of homogeneous bsAbs using traditional techniques, hybrid hybridomas and chemical cross-linking, has limited their wider application as therapeutic reagents^5^; however, advanced design of formats have facilitated the production of homogeneous bsAbs. Numerous bsAb formats, ranging from IgG-like molecules to small molecules, such as diabodies (Dbs)^6^, single-chain Dbs (scDbs)^7^, tandem single-chain variable fragments^8^, and other derivatives^9^, have been reported. Some of these small bsAb formats can be produced in bacterial expression systems and are used as building blocks to design more functional molecular formats, such as that of the human Fc fusion^10^.

**Figure 1:**
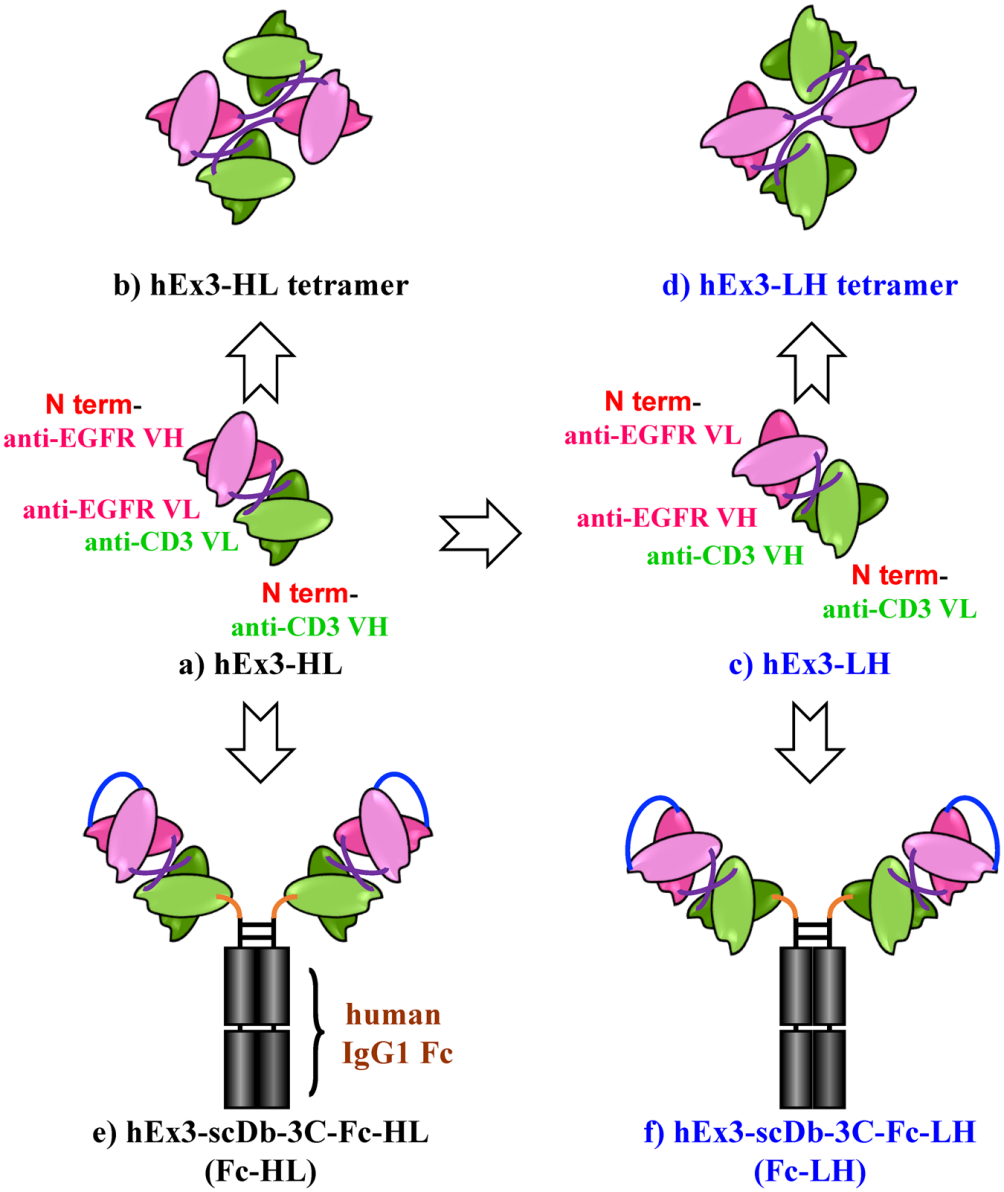
Schematic diagrams of bsAb formats evaluated in this study. Bispecific diabody that targets epidermal growth factor receptor and CD3 (**a**) and its tetramer that emerged during the preparation (**b**), domain order rearranged format, in which both components were in the VL-VH order (**c**,**d**), and Fc fusion formats (**e**,**f**).

We previously developed a bsAb and constructed a functional humanized bsDb that targets epidermal growth factor receptor (EGFR) and CD3 (hEx3-HL) (Fig. 1A)^11^. We also reported the substantial intense cancer growth inhibition effects of fractionated bsDb tetramers that emerged during the preparation of hEx3-HL (Fig. 1B)^12^. Further, marked enhancement in the cytotoxicity of hEx3-HL was achieved by rearranging the domain order of the V domain, particularly LH type (hEx3-LH) (Fig. 1C,D), in which both components were in the VL-VH order and exerted the strongest anti-tumor activity^13^. Finally, using bsDbs as building blocks, we generated their Fc fusion formats and confirmed any additional enhanced effects (Fig. 1E,F)^14,15^. In contrast, affinity maturation is an important strategy for improving antibody function. In fact, reductions in the affinity of anti-EGFR antibody 528, used in our study were observed after humanization of the antibody^16^. Thus, we isolated high-affinity humanized 528 (h528) VH mutants using a phage display method; and increased cancer growth inhibition was observed by integrating these mutants into hEx3-HL^17^. However, we have not applied the approaches used to design hEx3-HL, in other functional bsAb formats. Further, we recently isolated high-affinity h528 VL mutants^18^.

Here, to create additional functional bsAbs, we integrated the high-affinity h528 VH and VL mutants into hEx3-LH and Fc-fused hEx3-Dbs. The affinity of hEx3-LH was successfully improved by introducing mutations, which enhanced the cytotoxicity of the bsAbs *in vitro* and *in vivo*. However, there were some limitations to the affinity maturation of bsDb caused by integrating mutants isolated in the Fv form. In Fc-fused hEx3-Dbs, introducing mutations enhanced *in vitro* cytotoxicity, but not *in vivo*. Although a more sensitive *in vivo* model is needed to evaluate these molecules, we successfully developed a highly potent small bsDb by integrating a high-affinity mutant; particularly, the hEx3-LH mutant tetramer showed comparable *in vivo* therapeutic effects to Fc-fused hEx3-Dbs.

## Results

### Preparation of hEx3-LH mutants and evaluation of affinity

To improve the function of hEx3-LH, we integrated it with high-affinity mutants and evaluated the affinity of the modified antibodies. We recovered the reductions in the affinity of 528 caused by humanization using phage display and isolated high-affinity mutants. First, we isolated a single h528 VH mutant, HY52W, and then obtained a higher affinity mutant, 2HH11, with three additional mutations compared to those of HY52W^17^. Finally, based on 2HH11, we isolated much higher affinity h528 VL mutants, 2L1 and 2L6 that contain three and two mutated residues, respectively^18^. Here, we integrated the mutants into hEx3-LH and prepared dimers using the *Escherichia coli* expression system described in the Materials and Methods. To investigate the interaction between soluble EGFR (sEGFR) and hEx3-LH mutants, we determined the binding kinetics using immobilized sEGFR and surface plasmon resonance (SPR) spectroscopy (Table 1). Increased affinity was observed in LH-HY52W; however, additional improvements were not observed with the other hEx3-LH mutants, although higher affinity mutants of h528 Fv (2HH11, 2HH11-2L1, and 2HH11-2L6) were used in their construction. The affinity of hEx3-LH was improved, but it was found some limitation in increasing affinity through integrating mutants isolated in the Fv form.

**Table 1:** Binding parameters of bsDbs.

	sEGFR		
	*k*_on_ (×10^5^ M^−1^s^−1^)	*k*_off_ (×10^−3^ s^–1^)	*K*_A_ (×10^7^ M^−1^)
hEx3-HL	0.93	2.0	4.6^a^
hEx3-LH	0.73	3.6	2.0^a^
LH-HY52W	2.2	0.64	34
LH-2HH11	1.4	0.50	29
LH-2HH11-2L1	2.4	0.56	43
LH-2HH11-2L6	2.6	0.65	41
E2x3-LH	8.2	4.3	19^a^

^a^Data from our previous report^19^.

### Effects of mutations in hEx3-LH on cancer growth inhibition

**Figure 2:**
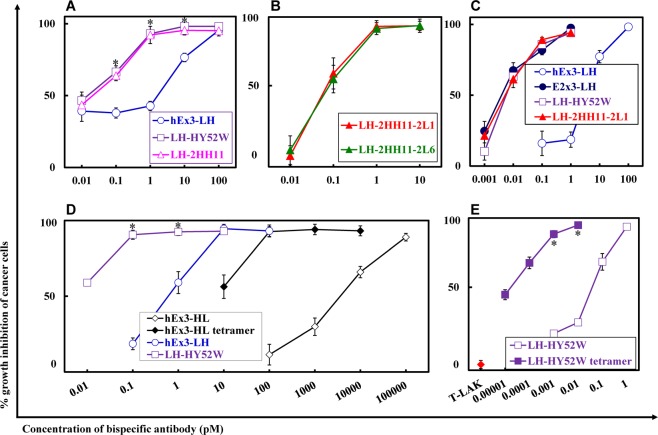
Growth inhibition of epidermal growth factor receptor (EGFR)-positive TFK-1 cells by each bispecific diabody (bsDb). Lymphokine-activated killer cells with T-cell phenotype (T-LAK) cells were added to cancer cells at a ratio of 2.5:1 (**B,C**) and 5:1 (**A**,**D**,**E**). Data are presented as the mean ± 1 SD and are representative of at least two independent experiments. *, Significant (P < 0.05) difference between LH-HY52W and hEx3-LH (**A**,**D**), and LH-HY52W tetramer and LH-HY52W (**E**). Statistical analysis was carried out using the Student’s *t* test.

To evaluate the influence of the increase in affinity, mediated by the mutations, on the inhibition of human carcinoma cell growth, we analyzed the four fractionated hEx3-LH mutant dimers in cell proliferation colorimetric assays. Enhanced cytotoxicity was observed for LH-HY52W (Fig. 2A); introduction of a single mutation effectively improved the function of hEx3-LH. However, consistent with the results of affinity evaluation, hEx3-LH mutants with additional mutations in LH-HY52W did not show improvement (Fig. 2A–C). The hEx3-LH mutants showed comparable effects to E2x3-LH (Fig. 2C), which was previously reported to have greater effects than hEx3-LH, but contains mouse anti-EGFR sequences unlike hEx3-LH^19^. We also observed the cancer growth inhibition effects of fractionated bsDb tetramers during the preparation of Db^12^. Here, we fractionated the tetramer of LH-HY52W, and as expected it had the highest cytotoxicity (Fig. 2D,E). These results demonstrate that introducing a high-affinity single point mutation enhances the cytotoxicity of hEx3-LH.

### Preparation and functional evaluation of Fc-fused hEx3-Db

**Figure 3:**
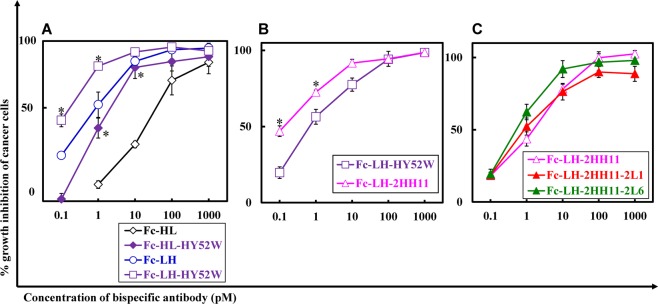
Growth inhibition of epidermal growth factor receptor (EGFR)-positive TFK-1 cells by each Fc-fused bispecific diabody (bsDb). Lymphokine-activated killer cells with T-cell phenotype (T-LAK) cells were added to TFK-1 cells at a ratio of 5:1 (**A**) and 3:1 (**B**). Peripheral blood mononuclear cells (PBMCs) were added to TFK-1 cells at a ratio of 40:1 (**C**). Data are presented as the mean ± 1 SD and are representative of at least two independent experiments. *Significant (P < 0.05) difference between Fc-HL-HY52W and Fc-HL, and Fc-LH-HY52W and Fc-LH (**A**), Fc-LH-2HH11 and Fc-LH-HY52W (**B**). Statistical analysis was carried out using the Student’s *t* test.

To create more functional bsAbs, we integrated high-affinity mutants into Fc-fused hEx3-Db, Fc-HL, and Fc-LH and prepared monomers using the Chinese hamster ovary (CHO) expression system as described in the Materials and Methods. Affinity was increased by introducing mutations according to SPR analysis; however, reliable kinetic values were not obtained because the intrinsic affinity of Fc-fused bsDbs is high, since the avidity effects caused by bivalent binding exceeds the limit of detection of the instrument. To evaluate the growth inhibition effects of Fc-fused hEx3-Db mutants, we analyzed cell proliferation in colorimetric assays as conducted for hEx3-Dbs. Introduction of the HY52W mutation also increased the cytotoxicity of both Fc-HL and Fc-LH (Fig. 3A). For Fc-LH, Fc-LH-2HH11 showed a slight further improvement compared to Fc-LH-HY52W (Fig. 3B). The cytotoxicity in the presence of unstimulated peripheral blood mononuclear cells (PBMCs) is important when considering the therapeutic potential of bsAbs. When PBMCs were used as effector cells, Fc-LH-2HH11 also inhibited cancer cell growth effectively, but further improvements were not observed when additional high-affinity VL mutants, Fc-LH-2HH11-2L1 and Fc-LH-2HH11-2L6, were integrated (Fig. 3C).

### *In vivo* antitumor effects of hEx3-LH mutants

**Figure 4:**
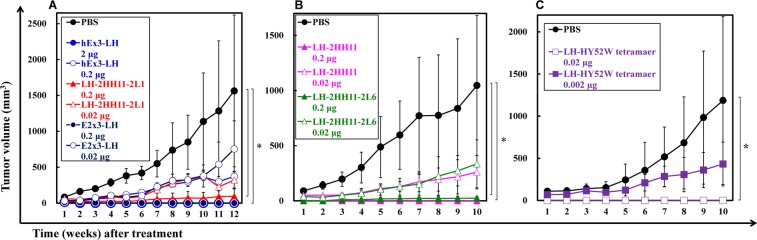
*In vivo* antitumor effects of bispecific diabodies (bsDbs). The mixture of 1.0 × 10^7^ T-LAK cells and 5 × 10^6^ TFK-1 cells was injected subcutaneously into the dorsal thoracic wall of female, 6-week-old severe combined immunodeficient (SCID) mice. Then, five mice per group were treated intravenously with bsAb or PBS at the indicated doses starting at 1 h after TFK-1 inoculation, and treatment was repeated once daily for 3 consecutive days. Data are shown as the median tumor volume (bar, SEM) in each treatment group. *Significant (*P* < 0.05) difference between PBS and 0.2 µg of LH-2HH11-2L1 or 0.2 µg of E2x3-LH (**A**), PBS and 0.2 µg of LH-2HH11 or 0.2 µg of LH-2HH11-2L6 (**B**), PBS and 0.02 µg of LH-HY52W tetramer (**C**). Statistical analysis was carried out using the two-way ANOVA test.

To compare *in vivo* antitumor effects of the hEx3-LH and hEx3-LH mutants, we transplanted mixtures of human bile duct carcinoma (TFK-1) cells and lymphokine-activated killer cells with the T-cell phenotype (T-LAK) cells into severe combined immunodeficient (SCID) mice, which were then treated for four days with bsDbs. Compared to the phosphate-buffered saline (PBS) controls, treatment with 2 µg of hEx3-LH markedly inhibited tumor growth in SCID mice; however, 0.2 µg of hEx3-LH showed only moderate growth inhibitory effects (Fig. 4A). In contrast, 0.2 µg of hEx3-LH mutants still inhibited tumor growth effectively (Fig. 4A,B). Consistent with the *in vitro* results, hEx3-LH mutant (2HH11-2L1) showed comparable effects to E2x3-LH (Fig. 4A), and there were no major differences between hEx3-LH mutants with additional mutations in LH-HY52W (2HH11 and 2HH11-2L6) (Fig. 4B). Even at a dose of 0.02 µg, in which hEx3-LH mutants only exhibited moderate antitumor effects, the LH-HY52W tetramer inhibited tumor growth; this effect was greatly reduced at a dose of 0.002 µg (Fig. 4C). These results demonstrate that the high-affinity mutations inhibited tumor growth in an *in vivo* tumor model.

### *In vivo* antitumor effects of Fc-fused hEx3-Db

**Figure 5:**
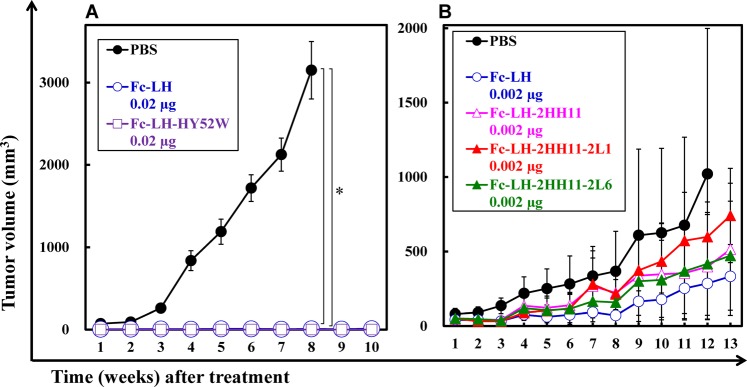
*In vivo* antitumor effects of Fc-fused bispecific diabodies (bsDbs). The mixture of 1.0 × 10^7^ T-LAK cells and 5 × 10^6^ TFK-1 cells was injected subcutaneously into the dorsal thoracic wall of female, 6-week-old severe combined immunodeficient (SCID) mice. Then, five mice per group were treated intravenously with bsAb or PBS at the indicated doses starting at 1 h after TFK-1 inoculation, and treatment was repeated once daily for 3 consecutive days. Data are shown as the median tumor volume (bar, SEM) in each treatment group. *Significant (*P* < 0.05) difference between PBS and Fc-LH or Fc-LH-HY52W (**A**). Statistical analysis was carried out using the two-way ANOVA test.

To evaluate the *in vivo* antitumor activity of Fc-fused hEx3-Db mutants, we used the transplanted mouse model used to examine the hEx3-LH mutants. Although 0.02 µg of both LH-Fc and LH-Fc-HY52W markedly inhibited tumor growth in SCID mice (Fig. 5A), none of the LH-Fc mutants tested and 0.002 µg LH-Fc showed intense antitumor effects (Fig. 5B). In the *in vivo* tumor models used in this study, further enhancements in antitumor effects were not observed by introducing high-affinity mutation. Fc-fused bsDbs have intrinsically high affinity because of their bivalent effects, which may explain these results.

## Discussion

Designing non-natural antibody formats is a practical method for developing highly functional next-generation antibody drugs, particularly for improving the therapeutic efficacy for cancer treatments. One important approach is the construction of bsAbs that simultaneously bind two different targets. This cross-linking can be applied to several therapeutic strategies for the treatment of cancer^20^; however, most bsAbs that have been designed redirect some effector cells mainly T cells toward cancer cells. BsAbs were the first non-natural antibody formats approved by the FDA; however, to date only one has been approved for the treatment of cancer. To accelerate the development of bsAb drugs, we studied the design of functional bsAbs.

In the present study, to construct highly functional bsAbs, we integrated high-affinity mutants previously isolated by phage display^17,18^ into a series of hEx3 bsAbs with specificity for EGFR and CD3^11–15^. Increased affinity was observed in LH-HY52W, resulting in cytotoxic enhancements *in vitro*, as expected (Table 1 and Fig. 2A), However, further increases in cytotoxicity were not observed for other hEx3-LH mutants, LH-2HH11, -2HH11-2L1, and -2HH11-2L6 (Table 1 and Fig. 2B,C). These mutants were selected in the form of Fv and showed higher affinity than HY52W^17,18^. The HY52W mutation may be critical for increasing the interaction between h528 Fv and EGFR and additional mutations may contribute specifically to the Fv form by stabilizing the VH and VL interactions. To further improve hEx3-LH, direct selection using the diabody form may be effective^21^.

In Fc-fused hEx3-Dbs, introduction of the HY52W mutation was also effective in increasing cytotoxicity (Fig. 3A). As observed with hEx3-LH, the effects were not enhanced by incorporating additional mutations, Fc-LH-2HH11-2L1 and -2HH11-2L6, although Fc-LH-2HH11 showed slightly greater effects than -HY52W. However, in *in vivo* tumor models, the cytotoxic effects of all mutants were comparable to that of LH-Fc (Fig. 5A,B). In contrast, consistent with the *in vitro* results, all hEx3-LH mutants showed greater antitumor effects than hEx3-LH, but similar levels to each other. Fc fusion has intrinsically high affinity because of its bivalency. We did not confirm the effect of introducing high-affinity mutations into the Fc fusion *in vivo*, and a more sensitive *in vivo* model is needed to evaluate these high-affinity molecules.

In contrast to classic bsAbs prepared by chemical conjugation and quadroma production^22–24^ or Fc-fused bsAbs such as Fc-LH, small bsAbs may allow for greater tissue penetration and higher target retention, and can be produced using cost-efficient microbial expression systems^25,26^. Hosts such as *E. coli*^6^ and *Pichia pastoris*^27^ have been used to produce small bsAbs; however, clinical-grade production in these hosts remains challenging. In fact, blinatumomab was produced in CHO cells, i.e., a mammalian expression system. *Brevibacillus choshinensis* is an attractive protein expression host, because it is a Gram-positive bacterium that secretes proteins directly into culture, shows low extracellular protease activity, produces no endotoxins, and is nonpathogenic^28–30^. Recently, we successfully expressed a small type of hEx3 bsAbs using *B. choshinensis* and demonstrated the utility of this organism as an expression host with high secretory productivity^31^. Thus, small bsAbs with high therapeutic potential are attractive candidates for the production of next-generation therapeutic antibodies.

However, the rapid blood clearance and monovalency of small bsAbs may limit their therapeutic applications; in fact, hEx3-LH mutants showed lower antitumor effects *in vivo* compared to LH-Fc (Figs. 4A,B, 5A,B). The length and amino acid composition of the linkers can be engineered to enable small bsAbs to assemble into multimers, such as tandem scDbs^32^ and bsDb tetramers^33^, with higher molecular weights and bivalency for target antigens. We also have found that hEx3 formed tetramers with high affinity and high cytotoxicity^12^. The multimerization of small antibody fragments may lead to improved pharmacokinetics^34^. In this study, LH-HY52W tetramers exhibited the highest *in vitro* growth inhibition among the small bsAbs (Fig. 2D,E) and was comparable to the *in vivo* anti-tumor effects of Fc-fused bsDbs (Figs. 4C, 5A). However, to increase the population of tetramers, additional engineering such as modification of the linker, similar to the modifications of the scFv multimer^35–37^, is required. bsDb tetramers are attractive molecules with high therapeutic potential.

We observed increased cytotoxicity with bsDbs *in vitro* and *in vivo* by integrating high-affinity mutants isolated in the Fv form. However, the effects of affinity maturation of bsDbs were limited, particularly with the Fc fusion formats. Affinity maturation by directly using each bsAb format by molecular-evolutional engineering techniques such as mammalian cell display^38^ and yeast display^39^ may effectively further improve these high-molecular weight molecules.

## Methods

### Construction of expression vectors for bsDbs

We previously described the construction of bacterial co-expression vectors for humanized anti-EGFR × humanized anti-CD3 bsDbs (hEx3) with different domain orders^13^: pRA-hEx3-HL for hEx3-HL, in which both chimeric single-chain components are in VH–VL order; pRA-hEx3-LH for hEx3-LH, in which both chimeric single-chain components are in VL–VH order. We also described the construction of a vector for mouse anti-EGFR × humanized anti-CD3 bsDbs (E2x3) using the V region of the approved therapeutic antibody cetuximab^19^: pRA-E2x3-LH for E2x3-LH in VL–VH order. The parental anti-EGFR antibody clones for hEx3s and E2x3-LH were 528 and 225, respectively^40^.

To recover the reductions in the affinity of humanized 528, we successfully isolated the high-affinity h528 VH mutant HY52W and HY52W-based 2HH11 by phage display^17^. In the present study, for further affinity maturation, the high-affinity h528 VL mutants 2L1 and 2L6 were isolated using 2HH11^18^, and to improve the function of hEx3-LH, these mutants were integrated. Bacterial co-expression vectors for hEx3-LH mutants were prepared by the overlap extension PCR method: pRA-LH-HY52W, pRA-LH-2HH11, pRA-LH-2HH11-2L1, and pRA-LH-2HH11-2L6 for LH-HY52W, LH-2HH11, LH-2HH11-2L1, and LH-2HH11-2L6, respectively.

### Construction of expression vectors for Fc-fused bsDbs

We previously described the construction of the mammalian expression vectors pcDNA-hEx3-scDb-3C-Fc-HL for hEx3-scDb-3C-Fc-HL^14^ and pcDNA-hEx3-scDb-3C-Fc-LH for hEx3-scDb-3C-Fc-LH^15^. To simplify the designations, hEx3-scDb-3C-Fc-HL and hEx3-scDb-3C-Fc-LH were renamed as Fc-HL and Fc-LH in this study. To improve the function of Fc-fused bsDbs, we also integrated high-affinity h528 mutants into them. Mammalian expression vectors for Fc-fused bsDbs were prepared by the overlap extension PCR method: pcDNA-Fc-HL-HY52W, pcDNA-Fc-LH-HY52W, pcDNA-Fc-LH-2HH11, pcDNA-Fc-LH-2HH11-2L1, and pcDNA-Fc-LH-2HH11-2L6 for Fc-HL-HY52W, Fc-LH-HY52W, Fc-LH-2HH11, Fc-LH-2HH11-2L1, and Fc-LH-2HH11-2L6, respectively.

### Preparation of bsDbs

The bsDbs were prepared by using the bacterial expression system we described previously^13^. Briefly, the constructs were expressed individually in *E. coli* strain BL21 Star (DE3) (Life Technologies, Carlsbad, CA, USA) and purified from the bacterial supernatant and periplasmic fractions. The constructs were also prepared from the intracellular soluble fraction by using BugBuster reagent (Merck KGaA, Darmstadt, Germany) according to the manufacturer’s instructions^34^. After purification by immobilized metal-affinity chromatography, gel filtration analysis (HiLoad Superdex 200-pg column 26/60, GE Healthcare Bio-Science, Piscataway, NJ, USA) was used to fractionate the dimers of each bsDb and tetramers of LH-HY52W. The column was equilibrated with PBS, and then purified bsDb was loaded onto the column at a flow rate of 2–2.5 mL/min.

### Preparation of Fc-fused bsDbs

The methods used for expression in CHO cells and purification of Fc-fused bsDbs have been described previously^41^. Briefly, the Fc-fused bsDbs were first purified on a protein A column (GE Healthcare). Gel filtration analysis (HiLoad Superdex 200 pg column 26/600, GE Healthcare) was conducted to fractionate the monomers of each bsAb. The column was equilibrated with PBS, and protein A-purified bsAb was loaded onto the column at a flow rate of 2–2.5 mL/min.

### Surface plasmon resonance spectroscopy

The interactions between sEGFR and bsDbs were analyzed by SPR spectroscopy (Biacore 2000, GE Healthcare). The methods for the expression and purification of sEGFR and the evaluation conditions have been described previously^16,19^. sEGFR was immobilized on the cells in a CM5 sensor chip to a maximum of 1389 resonance units. BIAevaluation software (GE Healthcare) was used to analyze the data. Kinetic parameters were calculated by global fitting analysis assuming a 1:1 Langmuir binding model.

### Cell lines

TFK-1 cells were used in the present study for consistency with our previous reports^11–15,17,19^. The TFK-1 cell line was established by our group^42^. TFK-1 cells were cultured in Roswell Park Memorial Institute 1640 medium supplemented with 10% fetal bovine serum, 100 U/mL penicillin, and 100 μg/mL streptomycin.

### *In vitro* killing assay

T-LAK cells were induced as described previously^43^. Briefly, PBMCs were cultured for 48 h at a density of 2 × 10^6^ cells/mL in medium supplemented with 100 IU/mL recombinant human interleukin-2 (Shionogi Pharmaceutical Co., Osaka, Japan) in a culture flask (A/S Nunc, Roskilde, Denmark) that was pre-coated with anti-CD3 monoclonal antibody (10 μg/mL). *In vitro* growth inhibition of the cancer cells was evaluated by MTS assay kit (CellTiter 96® AQueous Non-Radioactive Cell Proliferation Assay; Promega, Madison, WI, USA) as reported previously^43^. Basal activity of T-LAK cells changed depending on the lot of PBMCs. Thus, we performed MTS assay using different ratios of T-LAK cells to cancer cells for each experiment and indicated in the Figure legends.

### *In vivo* tumor models

The methods used to evaluate the *in vivo* antitumor properties of bsAbs have been previously described^19^. The experiments and procedures involving mice were reviewed and approved by the Committee on Ethics in Animal Experiments of Tohoku University and were performed under the Guidelines for Animal Experiments of Tohoku University according to the laws and notifications of the Japanese government.
